# Cell Membrane CD44v6 Levels in Squamous Cell Carcinoma of the Lung: Association with High Cellular Proliferation and High Concentrations of EGFR and CD44v5

**DOI:** 10.3390/ijms16034372

**Published:** 2015-02-18

**Authors:** Álvaro Ruibal, Pablo Aguiar, María Carmen Del Río, Matilde Isabel Nuñez, Virginia Pubul, Michel Herranz

**Affiliations:** 1 Molecular Imaging Group, Faculty of Medicine, University of Santiago Compostela, R/de San Francisco, s/n., Santiago de Compostela 15782, Spain; E-Mails: alvaro.ruibal.morell@sergas.es (A.R.); michel.herranz.carnero@sergas.es (M.H.); 2 Nuclear Medicine Department, University Hospital Santiago Compostela (CHUS), R/Choupana, s/n., Santiago de Compostela 15706, Spain; E-Mails: matilde.isabel.nunez@sergas.es (M.I.N.); virginia.pubul.nunez@sergas.es (V.P.); 3 Fundación Tejerina, C/José Abascal, 40, Madrid 28003, Spain; 4 General Lab, Hospital Arquitecto Marcide, Ferrol, A Coruña 15405, Spain; E-Mail: maria.del.carmen.del.rio.garma@sergas.es

**Keywords:** lung cancer, squamous lung carcinomas, CD44v6, CD44v5, EGFR, proliferation

## Abstract

Membranous CD44v6 levels in tumors and surrounding samples obtained from 94 patients with squamous cell lung carcinomas were studied and compared to clinical stage, cellular proliferation, membranous CD44v5 levels, epidermal growth factor receptor EGFR and cytoplasmatic concentrations of CYFRA 21.1. CD44v6 positive values were observed in 33/38 non-tumor samples and in 76/94 tumor samples, but there were not statistically significant differences between both subgroups. In CD44v6 positive tumor samples, CD44v6 was not associated with clinical stage, histological grade, ploidy and lymph node involvement, but significant association was found with high cellular proliferation. Likewise, CD44v6 positive tumors had significantly higher levels of EGFR and CD44v5. In patients with squamous cell lung carcinomas and clinical stage I, positive CD44v6 cases were associated with the same parameters. Furthermore, positive CD44v5 squamous tumors were associated significantly with histological grade III and lower levels of CYFRA21.1. Our findings support the value of CD44v6 as a possible indicator of poor outcome in patients with squamous lung carcinomas.

## 1. Introduction

CD44 is a surface adhesion molecule. It consists of a transmembrane glycoprotein expressed in many normal tissues by cells of different origin and involved in some physiological processes such as cell-cell and cell-matrix adhesion, hematopoiesis, lymphocyte homing and activation, and tumor dissemination [1,2]. CD44 exists in a standard form (CD44s) and in multiple isoforms, generated by alternative splicing of at least 10 variant exons (CD44v) encoding parts of the extracellular domain and related with some features of the tumor evolution [3]. One of them is CD44v6, associated with the metastatic potential of some human malignant tumors, regulating tumor invasion, progression and metastasis [4,5,6,7]. Classical studies in non-small cell lung cancers have demonstrated that CD44v6 expression was higher in squamous than in adenocarcinoma subtype [8,9,10] and that it was associated with a poorer survival, included stage I [3]. Nevertheless, its association with lymphatic or vascular invasion, as well as the outcome is not unanimous [8], whereas there was no any relation neither with histological grade nor clinical stage [10].

In the last years the clinical interest for this molecule has increased. Recently, Zhao *et al.* [11] and Jiang *et al.* [12] after two meta-analysis, showed that CD44v6 expression in non-small cell lung cancer (NSCLC) is associated with squamous subtype, lymph node metastasis, and a poor survival, considering a new prognostic marker in patients with these lung carcinomas. Furthermore, Sun *et al.* [13] studied the behavior of CD44v6 and osteopontin, gene involved in metastatic process of malignant tumors binding to CD44v6 and integrin, and considered that both parameters were independent predictors for poorer overall survival and disease free survival. These facts and the lack of papers differentiating the most important subtypes in non-small cell lung cancer NSCLC, led us to study the possible associations of CD44v6 with other clinical and biological parameters only in patients with squamous cell lung cancer.

## 2. Results and Discussion

Normal lung tissues had higher levels of cathepsin D than tumor tissues (*r*: 8.9–1332; median 74.6 pmol/mg prot. *vs.* *r*: 7.7–567; median 38.8; *p*: 0.0019), while tumor tissues had higher levels of CD44s, CD44v5, CD44v6, EGFR, CA125 and neuron specific enolase (NSE). (See Table 1).

CD44v6 positive values (>5 ng/mg prot.) were observed in 33/38 non-tumor samples (87%) and in 76/94 tumor samples (81%). There were not statistically significant differences between both subgroups. CD44v6 values correlated significantly with CD44v5 levels both in non-tumor samples (*r*: 0.78) and in tumor samples (*r*: 0.89). Likewise, only in tumoral tissues, there were statistically significant correlations between CD44v5 and CD44s (*r*: 0.64), between CD44v6 and CD44s (*r*: 0.57) and between CD44v6 and cytosolic hyaluronic acid concentrations (*r*: 0.26).

Finally, it should be mentioned that CD44v6 levels obtained in squamous tumor samples (range: 5.1–1395; median 68.2) were significantly higher (*p*: 0.00006) than those observed in 34 adenocarcinoma tumors (*r*: 5.8–454; median 28.8 ng/mg prot.). Our findings were similar to those described by other authors as Miyoshi *et al.* [14], Wu *et al.* [15] and slightly higher than those reported by Eren *et al.* [8].

**Table 1:** Statistically significant differences in some biological parameters between normal and squamous cell lung tissues.

Parameter	Normal	Tumor Tissue	*p*
CD44s **	84.1–237 (127)	80.6–643 (153)	0.016
CD44v5 **	3.5–47.6 (22.7)	3.5–1080 (33.8)	0.001
CD44v6 **	8.6–100 (32.3)	5.1–1305 (68.2)	<0.001
EGFR *	2.7–45.8 (19.2)	1–394 (34.7)	<0.001
CA125 **	1–51.7 (8.4)	1–576 (12.8)	0.005
NSE **	56–657 (141)	4.5–2234 (267)	<0.001

Range (median); *: fmol/mg prot.; and **: ng/mg prot.

In the 76 CD44v6 positive tumor samples, CD4v6 doesn’t associate with clinical stage, histological grade, ploidy and lymph node involvement but significant association (*p*: 0.015) between high CD44v6 levels and high cellular proliferation (SP) (31/49 *vs.* 2/13) measured by S-phase was found (cut off: 14%, which represents, in our experience, the percentile 75th of all values obtained with more than 200 lung tumors. Positive CD44v6 tumors had higher levels of EGFR (*r*: 3.1–325; median 38.7 *vs*. *r*: 1–394; median 20.5 fmol/mg prot.; *p*: 0.0046), CD44v5 levels (*r*: 3.5–1080, median 42.2 *vs.* *r*: 6.9–45.8; median 17.5 ng/mg prot.; *p*: 0.0002), CA125, NSE and cytosolic hyaluronic acid concentrations (*r*: 50–22591; median 5154 *vs.* *r*: 817–9799; median 3101 ng/mg prot.; *p*: 0.008).

Same findings were observed also in patients with squamous cell lung carcinomas in clinical stage I: high SP (*p*: 0.021), higher CD44v5 (*p*: 0.001) and higher EGFR levels (*p*: 0.01) (see Table 2). It deserves to be outlined that positive CD44v5 squamous tumors were associated significantly (*p*: 0.021) with histological grade III and lower levels of CYFRA 21.1(*r*: 16.5–3306; median 119 *vs.* *r*: 113–3817; median 383 ng/mg prot.; *p*: 0.0081).

Other groups described an association between CD44v6 positivity and lymph node metastasis [14,15], poorly differentiated tumors, advanced clinical stage and poorer survival [12,15], being a prognostic factor with pTNM stage after multivariate analysis [15], even in stage I non-small cell lung [3]. Hirata *et al.* [3] described also a correlation between CD44v6 and proliferating cell nuclear antigen PCNA positive expression, another indicator of cellular proliferation. We know that cell proliferation is associated with a poor prognosis in some tumors and with better response to chemotherapy [16].

It has to be mentioned that EGFR levels in NSCLC samples are higher than those observed in normal lung tissues and they are related with a poor tumor differentiation and a high proliferation. Likewise, they are associated with a poorer prognostic and outcome in squamous lung tumors [17,18]. Now, we know the clinical interest of the mutation in EGFR gene and the different behavior of these alterations according to the age of the patient [19]. CYFRA 21.1, which reflects fragments of cytokeratin 19, is a good serum tumor marker for the diagnosis of lung cancer, specially the squamous subtype in addition to computerized tomography, is associated with poor outcome in patients with NSCLC treated with adjuvant chemotherapy or erlotinib [20,21,22,23] and it is an useful tool for metastasis detection in lung cancer patients without symptoms of metastasis in patients with those lung tumors [24]. Nevertheless, the CYFRA 21.1 behavior in lung tumor tissues is opposite to what happens in serum; so, the weak expression of CK19, as determined by immunostaning intensity in tumor tissues and a high serum concentration of CYFRA 21.1 was a significant predictor of poorer disease-specific survival in human lung squamous cell carcinoma [25].

**Table 2:** Distribution of the biological parameters with statistically significant differences in squamous cell carcinomas of the lung (global and stage I) classified according to CD44v6 positivity.

Parameter	CD44v6+	CD44v6−	*p*
**Global**			
EGFR *	3.1–325 (38.7)	1–394 (20.5)	0.005
CD44v5 **	3.5–1080 (42.2)	6.9–45.8 (17.5)	0.0002
**Stage I**			
EGFR *	3.1–215 (35.8)	1–394 (21.3)	0.010
CD44v5 **	9.5–1080 (45.3)	6.9–45.8 (17.6)	0.001

Range (median); *: fmol/mg prot.; and **: ng/mg prot.

## 3. Experimental Section

### 3.1. Study Design

Membranous CD44v6 levels in tumors and surrounding samples were obtained from patients with squamous cell carcinomas of the lung.

### 3.2. Subjects

The study group was 94 patients (85 male and 9 female; age: 36–72; median 62 years) with squamous cell lung carcinomas. According to clinical stage, the patients were classified as follows: I = 69; II = 5; III = 20 cases. Likewise, 38 lung normal samples were removed from a region of surrounding tissue at least 2 cm away from the tumors. They were macroscopically free from neoplastic growth. A study group of 34 patients with adenocarcinoma tumors were also considered for comparison.

### 3.3. Blood Samples and Methods

Lung carcinoma tissue samples were obtained at the time of surgery. Immediately after surgical resection, samples were processed for pathological examination while the remainder tissue was washed with cold saline solution, divided in aliquots, rapidly transported on ice to the laboratory (−70 °C) pending biochemical studies. The specimens obtained from neoplastic tissues were pulverized with a microdismembrator (Braun Biotech International, Melsungen, Germany) at −70 °C and homogenized in Tris-hydrochloride buffer (10 mM of Tris, 1.5 mM of EDTA, 10% glycerol, 0.1% of monothioglycerol). Homogenates, kept at 4 °C, were centrifuged at low speed (800× *g* for 10 min, at 4°C), and the supernatant was ultracentrifuged at 100,000× *g* for 60 min, at 4 °C. We obtained a supernatant containing the cytsol and a precipitate with the membranes.

CD44v6 and CD44v5 were assayed in cell surface membranes using an enzymoimmunoassay from Bender MedSystems (Vienna, Austria) with two monoclonal antibodies. The lowest limit of sensitivity was 0.13 ng/mL for both CD44v6 and CD44v5. The intraassay variation coefficient of CD44v6 for a mean value of 0.52 and 6.4 ng/mL were 8.6% and 5.1% respectively and the intraassay variation coefficient for a mean value of 3.47 and 9.2 ng/mL were 9.3% and 8.4% respectively. The intraassay variation coefficient of CD44v5 for a mean value of 0.86 and 5.2 ng/mL were 8.6% and 5.0% respectively and the interassay variation coefficient for a mean value of 0.95 and 7.4 ng/mL were 9.3% and 7.4% respectively. Each sample was dossified by duplicate. The cut-off of positivity was established in 5 ng/mg prot. for CD44v6 and 3 ng/mg prot. for CD44v5 respectively.

Epidermal growth factor receptor (EGFR) was assayed in cell surface membrane using a radioligand method (ViennaLab, Vienna, Austria) with a lowest limit of sensitivity of 1 fmol/mg prot. CYFRA 21.1 was assayed using an immunoradiometric assay (CIS International, Gif sur Yvette, France) with two monoclonal antibodies (KS19.1 and BM 19.21) and with a lowest limit of sensitivity of 0.1 ng/mL, CD44s with an enzymoimmunoassay (Bender MedSystems, Vienna, Austria) with a lower limit of sensitivity of 0.42 ng/mL, Hyaluronic Acid using a Radioligand method from Pharmacia (Upjohn, Sweden) with a lower limit of sensitivity of 1 ng/mL, CA125 with an immunoradiometros assys (Centocor, Malvern, PA, USA) with a lower limit of sensitivity of 0.4 U/mL, NSE with an immunoradiometris assay (CIS BioInternational, France) with two monoclonal antibodies and a lower limit of sensitivity of 0.3 ng/mL, and cathepsin D was assayed using an immunoradiometric assay (CIS BioInternational) with two monoclonal antibodies and a lower limit of sensitivity of 20 pmol/mL.

All results were referred to mg of protein measured by Bradford method [26]. DNA ploidy and proliferative activity were evaluated by flow cytometry (Becton Dickinson, San Jose, CA, USA), on nuclei obtained from fresh samples and stained with propidium iodide, and calculated with the CellFit software program (Becton Dickinson), according to the DNA Cytometry Consensus Conference recommendations [27].

### 3.4. Statistics Analysis

After analyzing the distribution of CD44v6 and CD44v5 values by the Kolmogorov–Smirnov test, non-parametric rank methods were used because those parameters that did not follow a normal distribution. CD44v6 and Cd44v5 levels content were expressed as median and range. Comparison of the CD44v6 levels between different subgroups groups was made with the Mann–Whitney and Kruskal–Wallis tests. Correlations between continuous variables were calculated by the Spearman test. Differeces in percentages were calculated with the v2 test with Yates correction, if necessary. A *p*-value < 0.05 was considered as statistically significant.

## 4. Conclusions

Our results showed that CD44v6 positive values were observed in 80% of squamous tumor samples and were associated with high cellular proliferation, high levels of EGFR and high CD44v5 concentrations in cell membranes. Furthermore, positive CD44v5 squamous tumors were associated significantly with histological grade III and lower levels of CYFRA 21.1 in cytosols.

Our findings support the value of CD44v6 as an indicator of poor outcome in patients with squamous lung carcinomas.
